# Expression of MicroRNA-155 and its associations with EBV serological markers and inflammatory cytokines in young lymphoma patients with evidence of active EBV infection

**DOI:** 10.3389/ebm.2026.10869

**Published:** 2026-02-03

**Authors:** Lezan Medhat Mohammed, Payman S. Ali, Ali Qasim Taha, Luay M. Mohammad

**Affiliations:** 1 Medical Technical Institute/Kirkuk, Northern Technical University, Ministry of Higher Education and Scientific Research, Kirkuk, Iraq; 2 Department of Nurse, Medical Technical Institute/Kirkuk, Northern Technical University, Ministry of Higher Education and Scientific Research, Kirkuk, Iraq; 3 Department of Public Health, Kirkuk Health Directorate, Iraqi Ministry of Health (MOH), Kirkuk, Iraq

**Keywords:** cytokines (IL-18 and IL-32), Epstein–Barr virus, Iraq, microRNA-155, young lymphoma patients

## Abstract

The Epstein–Barr virus (EBV) is implicated in several lymphoproliferative disorders, particularly among children and adolescents who frequently experience primary EBV infection. MicroRNA-155 (miR-155), an oncogenic and immunoregulatory molecule, is known to participate in EBV-related immune modulation; however, its expression profile and relationship with EBV serological markers and inflammatory cytokines in young lymphoma patients remain insufficiently characterized. This cross-sectional observational study included 80 participants, comprising 40 young lymphoma patients with serological evidence of active EBV infection and 40 healthy controls. Serum EBV IgM and IgG levels were measured using ELISA, as were IL-18 and IL-32 concentrations, while serum miR-155 levels were quantified using qRT-PCR with an absolute quantification approach. The mean age of participants was 13.19 ± 2.51 years, and 55% were male. Serum miR-155 levels were significantly higher in lymphoma patients compared with controls (median: 1.13 vs. 0.43 ng/mL; p = 0.012). Elevated miR-155 expression was significantly associated with EBV IgM positivity (p < 0.001), IL-18 (p = 0.001), and IL-32 (p < 0.001). In multivariate logistic regression analysis, IL-32 positivity emerged as a strong independent predictor of elevated miR-155 levels (AOR = 19.02, p = 0.001). Receiver operating characteristic curve analysis demonstrated good discriminative performance of miR-155 (AUC = 0.87), with 87% sensitivity and 90% specificity at a cutoff value of ≥1.11 ng/mL. These findings indicate that serum miR-155 is significantly elevated in young lymphoma patients with serological evidence of active EBV infection and is statistically associated with inflammatory cytokines, particularly IL-32. miR-155 may represent a promising non-invasive biomarker reflecting EBV-related immune activation, although tissue-based EBV confirmation and mechanistic studies are required to establish causality.

## Impact statement

This study addresses a clinically relevant gap in understanding how Epstein–Barr virus–related immune activity is reflected in young lymphoma patients. While microRNA-155 has been widely studied in adult malignancies and experimental models, its circulating expression profile in pediatric and adolescent lymphoma patients with active EBV serology has remained poorly defined. By demonstrating that serum microRNA-155 is significantly elevated in affected patients and closely associated with inflammatory signaling—particularly interleukin-32—this work advances the field by linking viral serological activity to a measurable, non-invasive biomarker. These findings provide new insight into the interaction between EBV infection and immune-mediated pathways in early-onset lymphoma. Importantly, this study supports the clinical relevance of serum microRNA-155 as a candidate biomarker for future risk stratification and monitoring in EBV-associated lymphoproliferative disorders, thereby informing both translational research and clinical investigation.

## Introduction

The Epstein-Barr virus (EBV) is a prevalent gamma-herpesvirus that infects more than 90% of people globally. It primarily targets B-lymphocytes and establishes lifelong latency within these cells. EBV is recognized as the first oncogenic pathogen linked to various cancers [1].

EBV contributes to the development of several lymphoproliferative disorders, including Hodgkin’s lymphoma, Burkitt lymphoma, diffuse large B-cell lymphoma, and post-transplant lymphoproliferative diseases. In these cases, weakened immune surveillance permits unchecked proliferation of B-cells [2–4]. EBV infections usually occur during childhood and are often asymptomatic. Following the initial infection, the virus enters a dormant state, with memory B-lymphocytes becoming infected and establishing latency. This process involves the expression of latent genes and changes in the host’s immune responses in humans [5, 6].

MicroRNA-155 (miR-155), are encoded by the B-cell integration cluster gene (BIC) and play a crucial role in regulating inflammation, T-cell proliferation, and apoptosis [7]. Dysregulation of miR-155 is associated with various cancers [8].

MiR-155 is an upregulated microRNA in EBV-associated malignant B-lymphocytes, and the EBV genes LMPI and EBNA2 promote the expression of microRNA-155 through enhancer elements. This upregulation is particularly necessary for the proliferation of EBV-infected B-lymphocytes [9]. The miR-155 influences the production of both anti-inflammatory and pro-inflammatory cytokines [10].

Additionally, the expression of immune checkpoints enhances EBV infection, leading to the release of immunosuppressive cytokines and the efflux of regulatory T cells in the tumor microenvironment [11]. Furthermore, interleukin-18 (IL-18) stimulates the expression of interferon-gamma, which is a key cytokine for controlling viral infections [12]. Moreover, interleukin-32 (IL-32) is recognized as a pro-inflammatory cytokine produced by T lymphocytes, natural killer cells, monocytes, and epithelial cells. It has been emphasized that IL-32 plays a significant role in the pathophysiology of the disease [13].

The association between miR-155 and cytokine expression may indicate an underlying immune-regulatory interaction, as suggested by previous research, especially in pediatric populations where immune responses and microRNA regulatory networks are still evolving [10, 12–15]. However, these interactions were not directly assessed in the current study.

Children and adolescents exhibit immunological characteristics that are notably different from those of adults. They have a higher likelihood of experiencing primary EBV infections and possess a more dynamic immune environment that is regulated by developmental factors. Notably, microRNA-155 regulatory networks, which include those that control lymphocyte function, undergo ontogenetic changes. The expression patterns of microRNA-155 shift during lymphopoiesis and transition from childhood to adulthood [14–16]. These developmental changes can result in different baseline and responsive levels of microRNA-155, like miR-155, in younger individuals compared to adults. Such variations highlight the importance of investigating miR-155 expression specifically within pediatric cohorts of EBV-associated lymphomas. Our discussion of EBV-related mechanisms is based on previously published experimental studies; these regulatory interactions were not directly evaluated in the present cohort.

Despite increasing evidence of miR-155s involvement in EBV-related oncogenesis, few studies have focused on its expression in young patients with lymphoma and serological evidence of active EBV infection and its correlation with viral serological markers (IgM and IgG) and inflammatory cytokines. Understanding these relationships could offer valuable insights into the diagnostic and prognostic significance of miR-155 in early-onset lymphoma and may aid in identifying new therapeutic targets.

This study aimed to examine the expression levels of miR-155 in young patients with lymphoma who demonstrated serological evidence of active EBV infection, investigate its association with EBV IgM/IgG serological markers and inflammatory cytokines (IL-18 and IL-32), and evaluate its diagnostic potential.

## Materials and methods

### Study design and setting

This cross-sectional observational was carried out between November 2024 and May 2025 at the Teaching Oncology Hospital in Baghdad, Iraq. The objective of the study was to evaluate the expression of microRNA-155 (miR-155) and its relationship with Epstein-Barr virus (EBV) serological markers and inflammatory cytokines in young lymphoma patients with serological evidence of active EBV infection.

### Sample size determination

The required sample size was estimated using the formula for comparing two independent means [17]:

$$n=\frac{{\left(Z_{\alpha /2}+Z_{\beta }\right)}^{2}\cdot {2\sigma }^{2}}{\delta^{2}}$$

Where:

- Zα/2 = 1.96 (95% confidence)
- Zβ = 0.84 (80% power)
- δ = expected difference in miR-155 levels
- σ = pooled standard deviation

The sample size calculation utilized δ = 0.45 ng/mL as the expected difference in serum miR-155 levels between patients and controls, and σ = 0.62 ng/mL as the estimated pooled standard deviation based on previous studies of miR-155 in B-cell lymphomas [18, 19]. Using these parameters along with α = 0.05 and β = 0.20 determined a minimum required sample size of 39 participants per group. To account for potential missing data and maintain balanced groups, the sample was rounded to include 40 patients and 40 controls, totaling 80 participants.

Sensitivity analysis revealed that with 40 participants per group, the study could detect a minimum standardized effect size of 0.58 (considered a moderate effect) with 80% power.

### Participant selection criteria

- Inclusion Criteria
  - The study involved pediatric and adolescent patients aged 8 to 17, in line with established definitions of children and early adolescents with lymphoma. This age range was chosen due to the notable differences in immune responses, EBV infection patterns, and microRNA regulation compared to adults.
  - Diagnosed lymphoma based on clinical and hematological assessment.
  - Serological evidence of active EBV infection (positive EBV IgM and/or high EBV IgG titers).
- Exclusion Criteria
  - Recent infections other than EBV.
  - Co-infection with HIV, HBV, or HCV.
  - Autoimmune disorders or immunosuppressive therapy.
  - Previous malignancy.
  - Incomplete laboratory data.
- Controls
  Age- and sex-matched healthy individuals with negative EBV serology (IgM/IgG) and no history of malignancy.

### Data collection and laboratory procedures

#### Demographic and clinical data

Data regarding age, gender, and residency were gathered through structured interviews and medical records. Comprehensive histopathological subtype information (such as Hodgkin lymphoma, Burkitt lymphoma, and DLBCL) was not accessible for all patients in the clinical records; consequently, a subtype-specific analysis could not be conducted.

#### Serological testing

EBV serology was evaluated by detecting IgM and IgG antibodies using enzyme-linked immunosorbent assay (ELISA) kits from MyBioSource (USA), according to the manufacturer’s protocols (IgM: Cat# MBS029816; IgG: Cat# MBS285442). ELISA is a sensitive and validated method for diagnosing EBV serologically [20]. According to manufacturer data, both kits demonstrate sensitivities >95% and specificities >97%. Positivity was determined using the manufacturer-defined index values (≥1.1 = positive, 0.9–1.1 = equivocal, <0.9 = negative). All samples were analyzed in duplicate, and internal quality controls supplied with each kit were included on every assay plate. Laboratory personnel performing the ELISA assays were blinded to case/control status to minimize measurement bias.

#### Cytokine measurement

Serum levels of IL-18 and IL-32 were assessed using commercially available ELISA kits from MyBioSource (USA) (IL-18: Cat# MBS281497; IL-32: Cat# MBS580104). Classification as positive or negative was determined based on kit-specific cutoffs. These cytokines play a crucial role as inflammatory mediators in viral pathogenesis and lymphoproliferative disorders [21, 22]. The manufacturer reports assay sensitivities of 4.8 pg/mL for IL-18 and 9.38 pg/mL for IL-32, with specificities >95% and no notable cross-reactivity. All samples were run in duplicate with internal low- and high-control wells. Technicians conducting cytokine measurements were blinded to participant group allocation.

#### MicroRNA-155 expression quantification

Peripheral blood samples were collected in EDTA tubes, and total RNA was extracted using the miRNeasy Mini Kit (Qiagen, Germany). Quantitative reverse transcription polymerase chain reaction (qRT-PCR) was conducted using the miScript SYBR Green PCR Kit (Qiagen, Germany) according to the manufacturer’s instructions. Serum miR-155 levels were quantified using an absolute quantification method based on a standard curve provided by the kit, with results reported directly in ng/mL. Following MIQE recommendations [23, 24], an internal reaction control included in the assay ensured amplification quality; however, no endogenous reference gene was necessary for normalization. Raw Ct values were monitored to ensure amplification efficiency and reaction quality; however, they were not used for ΔCt or relative quantification because the assay followed an absolute quantification protocol. The optimal diagnostic cut-off value of ≥1.11 ng/mL was established based on ROC curve analysis.

### Statistical analysis

- The Shapiro-Wilk test was employed to evaluate the normality of the data.
- Mann-Whitney U (two groups) were utilized to compare miR-155 levels among different groups.
- Chi-square tests were conducted to examine associations between categorical variables and miR-155 positivity.
- Binary logistic regression was performed to identify predictors of elevated miR-155 levels, reporting both crude odds ratios (ORs) and adjusted odds ratios (AORs) with 95% confidence intervals.
- Receiver Operating Characteristic (ROC) curve analysis was carried out to evaluate the diagnostic performance of miR-155, with the Area Under the Curve (AUC) calculated to determine sensitivity and specificity.
- All analyses were performed using SPSS version 27, with a p-value of less than 0.05 considered statistically significant.

### Ethical considerations

The study received approval from the Technical Medical Institute, Kirkuk Northern Technical University, Ministry of Higher Education and Scientific Research, under approval IRB 73/21/11-2024. Written informed consent was obtained from the guardians of all participants.

## Results

**FIGURE 1:**
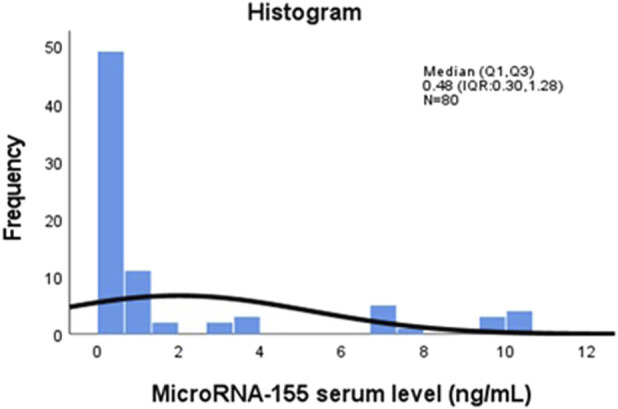
Distribution of serum MicroRNA-155 (miR-155) levels (ng/mL) among study participants (n = 80). The histogram displays the distribution of serum miR-155 concentrations measured by qRT-PCR. The x-axis represents serum miR-155 levels in ng/mL, and the y-axis represents the frequency of observations across the study population (n = 80).

A total of 80 participants were included in this study, comprising 40 young lymphoma patients with serological evidence of active EBV infection and 40 healthy controls matched for age and sex. The overall mean age of the study population was 13.19 ± 2.51 years (range: 8–17), with a median age of 13 years (IQR: 11–15), and 55% of the participants were male. Demographic, serological, and molecular characteristics were analyzed to assess serum microRNA-155 (miR-155) expression and its associations with EBV serological markers and proinflammatory cytokines. Figure 1 illustrates the distribution of serum miR-155 levels (ng/mL) among all participants. The histogram reveals significant variability in expression, with some individuals displaying much higher concentrations of miR-155. This visualization emphasizes the differences in miR-155 levels throughout the study population.

Table 1 presented a comparison of miR-155 expression between patients with lymphoma patients with serological evidence of active EBV infection and healthy controls. Lymphoma patients demonstrated a significantly median miR-155 expression level (1.13 ng/mL) compared to healthy controls (0.43 ng/mL), with a wide interquartile range and a maximum value exceeding 10 ng/mL. This difference was statistically significant (p = 0.012).

**TABLE 1:** Comparison of microRNA-155 expression levels between lymphoma patients with serological evidence of active EBV infection and healthy controls (n = 80).

Study groups	microRNA-155 (n = 80)				
	n	Median (Q1, Q3^a^)	Minimum	Maximum	P-value
Healthy controls	40	0.43 (0.29, 0.61)	0.11	0.77	0.012^b^
Patient groups	40	1.13 (0.32, 7.22)	0.12	10.53	​
Total	80	0.48 (0.30, 1.28)	0.11	10.53	

^a^ Q1 is quantile one and, Q3 is quantile three.

^b^ P value obtained from Mann-Whitney Test.

Table 2 summarizes the association between demographic and clinical variables and miR-155 positivity, defined as levels of ≥1.11 ng/mL. The dichotomous analysis (miR-155 positive vs. negative) revealed several significant relationships.

**TABLE 2:** Association of participant demographics and clinical characteristics by microRNA-155 positivity status (n = 80).

Variables	Total (%)	Negative microRNA155 n (%)^a^	Positive microRNA155 n (%)	P-value^b^
Total sample	80	60 (75.0)	20 (25.0)	​
Gender Female Male	36 (45.0) 44 (55.0)	28 (77.8) 32 (72.7)	8 (22.2) 12 (27.3)	0.004
Age group (years) (mean ± SD) 8–12 13–17	13.19 ± 2.51 32 (40.0) 48 (60.0)	13.27 ± 2.43 23(71.9) 37 (77.1)	13.00 ± 2.71 9 (28.1) 11 (22.9)	0.598
Residency Rural Urban	47 (58.8) 33 (41.3)	35 (74.5) 25 (75.8)	12 (25.5) 8 (24.2)	0.896
EVBIgM Negative Positive	41 (51.2) 39 (48.8)	41 (100.0) 19 (48.7)	0 (0.0) 20 (51.3)	<0.001
EVBIgG Negative Positive	66 (82.5) 14 (17.5)	51 (77.3) 9 (64.3)	15 (22.7) 5 (35.7)	0.308
Interleukin-18 Negative Positive	67 (83.8) 13 (16.3)	55 (82.1) 5 (38.5)	12 (17.9) 8 (61.5)	0.001
Interleukin-32 Negative Positive	41 (51.2) 39 (48.8)	40 (97.6) 20 (51.3)	1 (2.4) 19 (48.7)	<0.001

^a^ Frequency (%).

^b^ Chi-Square Tests.

Males exhibited a higher rate of miR-155 positivity (27.3%) compared to females (22.2%), and this difference was statistically significant (p = 0.004). A statistically significant association was also noted between EBV IgM positivity and miR-155 positivity (p < 0.001); all IgM-negative participants were miR-155 negative, whereas 51.3% of IgM-positive individuals were miR-155 positive.

Similarly, positivity for IL-18 and IL-32 showed statistically significant association with miR-155 positivity (p = 0.001 and p < 0.001, respectively). These findings suggest that elevated miR-155 levels are more closely related to EBV serological activity and inflammatory cytokine expression rather than demographic factors.

No significant associations were found for age or residency, indicating that the variability in miR-155 levels within this cohort is primarily influenced by immunological factors rather than demographic characteristics.

Table 3 presented a comparison of median miR-155 levels across subgroups (non-parametric analysis) and explores subgroup differences using continuous miR-155 values.

**TABLE 3:** Comparison of microRNA-155 expression levels across demographic and laboratory subgroups (n = 80).

Variables	microRNA-155 (n = 80)				
	n	Median (Q1, Q3)^a^	Minimum	Maximum	P-value
Gender Female Male	36 44	0.50 (0.25, 0.74) 0.45 (0.31, 1.49)	0.11 0.11	10.53 10.53	0.006^b^
Age group (years) 8–12 13–17	32 48	0.50 (0.32, 3.19) 0.47 (0.28, 0.75)	0.12 0.11	10.46 10.40	0.576
Residency Rural Urban	47 33	0.55 (0.30, 1.16) 0.47 (0.31, 0.74)	0.11 0.11	10.53 9.85	0.594
EVBIgM Negative Positive	41 39	0.43 (0.29, 0.61) 1.12 (0.32, 7.22)	0.11 0.11	0.77 10.53	0.004
EVBIgG Negative Positive	66 14	0.51 (0.30, 0.77) 0.39 (0.17, 3.19)	0.11 0.12	10.46 10.53	0.785
Interleukin-18 Negative Positive	67 13	0.44 (0.28, 0.72) 3.98 (0.67, 7.21)	0.11 0.11	10.46 10.53	0.008
Interleukin-32 Negative Positive	41 39	0.44 (0.30, 0.62) 0.77 (0.32, 7.20)	0.11 0.11	10.40 10.53	0.031

^a^ Q1 is quantile one and, Q3 is quantile three.

^b^ P value obtained from Mann-Whitney test.

In terms of gender, males exhibited significantly higher median miR-155 expression than females (p = 0.006).

A notable difference was observed between IgM-negative individuals (0.43 ng/mL) and IgM-positive individuals (1.12 ng/mL), with a p-value of 0.004.

Both interleukin-18 and IL-32 showed significant associations with miR-155 levels (p = 0.008 and p = 0.031, respectively), with particularly high miR-155 levels found in IL-18-positive individuals (median 3.98).

No significant differences were noted for age groups or residency, indicating that biological factors related to inflammation and infection are the primary drivers of miR-155 expression.

The binary logistic regression analysis for predictors of elevated miR-155 expression (≥1.11 ng/mL) is presented in Table 4. The model identified IL-32 positivity as a good predictor of high miR-155 levels, with a crude odds ratio (OR) of 18.01 and an adjusted odds ratio (AOR) of 19.02 (95% CI: 7.72–36.92, p = 0.001). This association indicates that IL-32 is closely linked to elevated miR-155 expression in this cohort.

**TABLE 4:** Predictors of elevated microRNA-155 expression among study participants: Logistic Regression Analysis (n = 80).

Variables	microRNA-155 (n = 80)				
	n	OR (95% CI)^a^	P-value	AOR (95% CI)^b^	P-value
Gender Female Male	36 44	Ref. 1.31 (0.47–3.67)	0.004	3.02 (0.63–11.43)	0.022
Age group (years) 8–12 13–17	32 48	Ref. 0.76 (0.27–2.11)	0.599	-	-
Residency Rural Urban	47 33	Ref. 0.93 (0.33–2.62)	0.933	-	-
EVBIgM Negative Positive	41 39	Ref. 1.05(0.49–3.59)	0.060	-	-
EVBIgG Negative Positive	66 14	Ref. 1.37 (0.54–3.53)	0.505	-	-
Interleukin-18 Negative Positive	67 13	Ref. 7.33 (2.03–26.38)	0.002	-	-
Interleukin-32 Negative Positive	41 39	Ref. 18.01 (4.74–30.56)	0.001	19.02 (7.72–36.92)	0.001

^a^ Crude odds ratio obtains from binary logistic regression.

^b^ Adjusted odds ratio obtains from Forward Stepwise (Likelihood Ratio).

IL-18 positivity was also significantly associated with higher miR-155 levels in univariate analysis (OR = 7.33, p = 0.002), but it did not remain significant in the final multivariable model. This may reflect biological overlap between IL-18 and IL-32 pathways or limited statistical power for multiple predictors.

**FIGURE 2:**
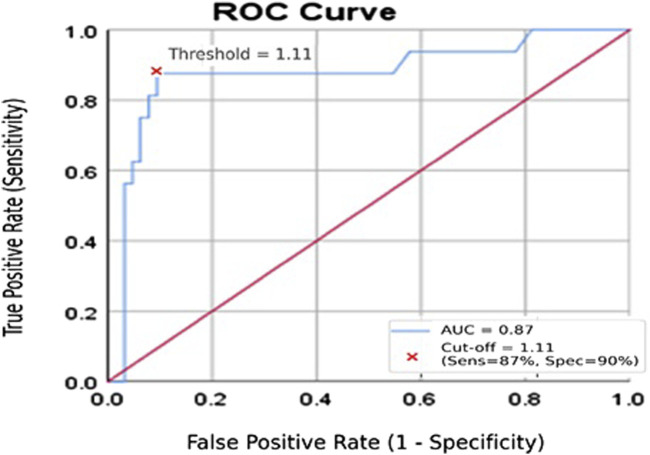
Receiver operating characteristic (ROC) curve for serum miR-155 in distinguishing lymphoma patients from healthy controls. The ROC curve depicts the diagnostic performance of serum miR-155 (ng/mL). The y-axis represents sensitivity, and the x-axis represents 1 − specificity. The analysis yielded an AUC of 0.87, indicating good discriminative ability.

The ROC curve illustrating the diagnostic performance of serum miR-155 in distinguishing lymphoma patients with serological evidence of EBV infection from healthy controls is shown in Figure 2. The analysis yielded an Area Under the Curve (AUC) of 0.87 (95% CI: 0.77–0.94, p < 0.001), demonstrating good ability to differentiate between the two groups. Bootstrap cross-validation using 1,000 resamples produced a bias-corrected AUC of approximately 0.87, confirming the robustness of the ROC estimate. Using a cut-off value of ≥1.11 ng/mL, miR-155 demonstrated a sensitivity of 87% and a specificity of 90%. These findings suggest that serum miR-155 may have potential utility as a non-invasive marker for identifying EBV-associated lymphoma risk, although validation in larger and independent cohorts is required.

## Discussion

This study examined serum microRNA-155 (miR-155) expression and its associations with Epstein–Barr virus (EBV) serological markers and proinflammatory cytokines (IL-18 and IL-32) in young lymphoma patients with evidence of active EBV infection. Our results indicate that serum miR-155 levels were significantly higher in patients than in healthy controls, suggesting that miR-155 may serve as a marker of EBV-related immune activity and systemic inflammation in this group.

In line with earlier reports that identify miR-155 as an oncogenic and immunoregulatory microRNA commonly upregulated in B-cell malignancies [7, 8], the median miR-155 concentration was significantly higher in the patient group. miR-155 has been linked to oncogenic processes through the regulation of pathways such as NF-κB, PI3K/AKT, and JAK/STAT [8, 9]. However, our findings are associative and do not establish a mechanistic role in the development of lymphoma.

A notable finding in this study was the statistically significant association between miR-155 overexpression and EBV IgM positivity, suggesting that elevated miR-155 may correlate with an active or recent EBV serological response. Experimental studies have demonstrated that EBV latent proteins—especially latent membrane protein-1 (LMP1)—activate NF-κB signaling and enhance the transcription of the BIC gene, which produces miR-155 [25, 26]. While these mechanisms are biologically plausible, this study cannot determine whether EBV directly induces miR-155 expression in tumor tissue, as gold-standard tissue-based confirmation methods (such as EBER-ISH or LMP1 IHC) were not employed.

Additionally, significant correlations were observed between elevated miR-155 levels and positivity for IL-18 and IL-32. Both cytokines play key roles in mediating inflammatory and antiviral immune responses. IL-18 enhances Th1-type immunity and activates MyD88-dependent NF-κB and STAT4 signaling [12, 27], while IL-32 stimulates the NF-κB, AP-1, and p38 MAPK pathways [13, 28, 29]. These signaling pathways have been implicated in the regulation of miR-155 expression. Although these findings suggest an association between inflammatory activation and increased miR-155 levels, they remain correlational, as functional experiments were not included in the current study design.

The associations observed between miR-155 expression and IL-18/IL-32 positivity may reflect previously described mechanistic interactions between cytokine-driven inflammatory signaling and the transcriptional regulation of miR-155. IL-18 activates NF-κB and STAT4 through MyD88-dependent pathways [12, 27], while IL-32 induces NF-κB, AP-1, and p38 MAPK activation [28, 29], and has been reported to enhance miR-155 expression via JAK1-dependent mechanisms [13]. These cytokine-driven pathways intersect with EBV latency-associated signaling: EBV latent proteins like LMP1 and EBNA2 activate NF-κB, AP-1, and JAK/STAT pathways [25, 26], and LMP1 can increase miR-155 transcription through BIC gene enhancers [9]. Collectively, these findings provide a biologically plausible framework that may explain the observed associations, although our study did not directly evaluate these mechanisms. Our findings are therefore consistent with previously described EBV- and cytokine-mediated regulatory pathways, but they do not establish mechanistic causality in this cohort. Future studies incorporating *in-vitro* LMP1/EBNA2 functional assays or analysis of EBV latency transcripts in tumor tissue are needed to experimentally validate these pathways.

**FIGURE 3:**
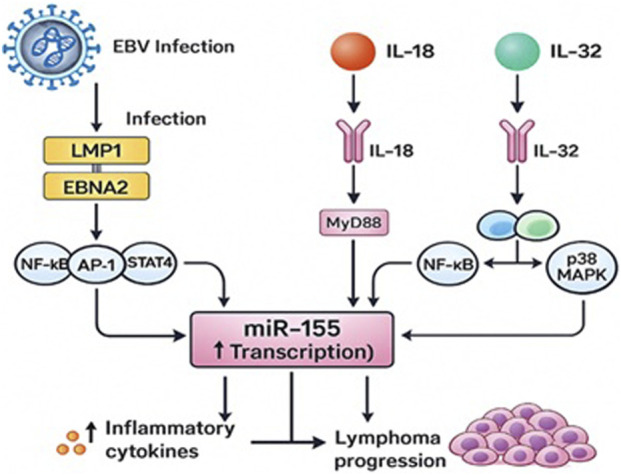
Proposed Mechanisms Linking EBV Infection, IL-18 and IL-32 Signaling, and miR-155 Upregulation in Young Lymphoma Patients.

The conceptual model presented in Figure 3 integrates previously reported mechanistic data regarding EBV-related signaling and cytokine-mediated regulation of miR-155. This diagram serves as a theoretical synthesis based on existing literature [9, 10, 25, 26, 29], rather than as direct evidence from this cohort.

Multivariate analysis identified IL-32 positivity as a good independent predictor of elevated miR-155 expression (AOR = 19.02), highlighting the robust association between IL-32 and miR-155 in this cohort. Male sex also remained independently associated with elevated miR-155 expression (AOR = 3.02, p = 0.022). While the biological basis is unclear, prior studies have reported gender-based differences in immune responses and microRNA regulation [30], which may contribute to this finding. However, given the modest sample size and the absence of lymphoma subtype data, this sex-related association should be interpreted cautiously and considered exploratory.

Receiver operating characteristic (ROC) analysis revealed that serum miR-155 has good discriminatory ability (AUC = 0.87) for distinguishing patients from controls. With a cutoff value of ≥1.11 ng/mL, miR-155 effectively separated the groups, in line with the sensitivity and specificity estimates derived from the ROC analysis. These results are consistent with earlier studies suggesting that miR-155 could be a potential biomarker for hematologic malignancies [7, 31]. However, because EBV status in tumor tissue was not validated and the cohort size was relatively small, diagnostic interpretations should be approached with caution.

Overall, the findings suggest that elevated serum miR-155 is linked to EBV serological activity and systemic inflammation, particularly with regard to IL-18 and IL-32, in young lymphoma patients. miR-155 may serve as a promising non-invasive biomarker indicative of EBV-related immune activation. However, confirmation of EBV in tissue samples and functional studies are necessary to establish whether EBV directly causes miR-155 dysregulation in lymphoma.

### Limitations

Although this study presents valuable findings, several limitations must be acknowledged. First, the cross-sectional design prevents assessment of temporal changes in miR-155 expression or its behavior during treatment or disease progression. Second, although the sample size was adequate to detect significant associations, it limits generalizability and reduces statistical power for subgroup analyses. Third, our cytokine analysis focused only on IL-18 and IL-32; evaluating a broader cytokine profile would have provided a more comprehensive view of the inflammatory microenvironment.

A major limitation is the absence of tissue-based confirmation of EBV tumor positivity. EBV status was determined solely by serology (IgM/IgG), as diagnostic tumor blocks were not available for EBER *in situ* hybridization, LMP1 immunohistochemistry, or EBV DNA PCR. Therefore, the cohort should be interpreted as lymphoma patients with serological evidence of active EBV infection, and not as confirmed EBV-positive lymphomas.

Another limitation is the lack of complete histopathological subtype information. Because subtype details were inconsistently documented in the clinical records, we were unable to stratify miR-155 expression according to lymphoma subtype, which may differ in biology and EBV association. Future studies should incorporate comprehensive subtype classification.

Finally, the mechanistic interpretations provided in this study are based on correlational findings. Although the observed associations are consistent with known EBV- and cytokine-related signaling pathways, functional experiments were not performed. Future longitudinal and mechanistic studies are required to validate these pathways and determine their clinical relevance. The ROC analysis was conducted in a single dataset without external validation; although bootstrap resampling supported the stability of the AUC, larger multicenter cohorts are required to validate the discriminative performance of miR-155.

### Conclusion

This study demonstrates that serum microRNA-155 (miR-155) is significantly elevated in young lymphoma patients with serological evidence of active EBV infection. miR-155 levels showed statistically significant association with EBV IgM positivity and with the proinflammatory cytokines IL-18 and IL-32, with IL-32 emerging as a good independent predictor of elevated miR-155. These findings suggest that heightened inflammatory signaling may contribute to the increased miR-155 expression observed in this cohort.

Serum miR-155 also demonstrated good discriminatory performance in distinguishing patients from healthy controls in this dataset. While this indicates potential utility as a non-invasive marker of EBV-related immune activation, it should not be interpreted as a diagnostic test because EBV tumor positivity was not confirmed using tissue-based methods. Larger studies with tissue confirmation and external validation are required to establish its diagnostic or prognostic value.

Overall, the results highlight a relevant relationship between EBV serological activity, inflammatory cytokines, and miR-155 expression in young lymphoma patients, supporting further investigation of miR-155 within EBV-associated immune and oncogenic pathways.

## Data Availability

The data generated in this study consist of targeted qRT-PCR measurements of a single microRNA (miR-155) and associated clinical variables from human participants. These data are not suitable for deposition in public gene-expression repositories and are not publicly available due to ethical and privacy restrictions. De-identified data supporting the findings of this study are available from the corresponding author upon reasonable request.
